# Aquatic therapy following arthroscopic rotator cuff repair enables faster improvement of Constant score than land-based therapy or self-rehabilitation therapy

**DOI:** 10.1186/s40634-022-00554-z

**Published:** 2023-01-13

**Authors:** Alec Cikes, Fayssal Kadri, Floris van Rooij, Alexandre Lädermann

**Affiliations:** 1 Synergy Medical Centre, Medbase Group, Lausanne, Switzerland; 2 Hirslanden, Bois Cerf Clinic, Lausanne, Switzerland; 3 ReSurg SA, Rue Saint Jean 22, 1260 Nyon, Switzerland; 4 Division of Orthopaedics and Trauma Surgery, La Tour Hospital, Meyrin, Switzerland; 5 Faculty of Medicine, University of Geneva, Geneva, Switzerland; 6 Division of Orthopaedics and Trauma Surgery, Department of Surgery, Geneva University Hospitals, Geneva, Switzerland

**Keywords:** Arthroscopic rotator cuff repair, Aquatic therapy, Land-based therapy, Self-rehabilitation therapy

## Abstract

**Purpose:**

To compare the clinical and functional outcomes of arthroscopic rotator cuff repair over a period of 2 years using three postoperative rehabilitation modalities: aquatic therapy, land-based therapy, and self-rehabilitation therapy. The null hypothesis was that aquatic therapy would provide no difference in Constant score compared to land-based therapy and self-rehabilitation therapy.

**Methods:**

A prospective study was performed on subjects scheduled for arthroscopic rotator cuff repair between 2012 and 2017 that complied with the following criteria: (i) small to medium sized symptomatic supraspinatus and/or infraspinatus tendon tears, (ii) low to moderate tendon retraction according to Patte, and (iii) fatty infiltration stage ≤2. Patients were allocated to perform either aquatic therapy, land-based therapy, or self-rehabilitation therapy for 2-4 months. Independent observers blinded to the study design collected Constant score, SSV, and patient satisfaction at 2 months, 3 months, 6 months, 1 year and 2 years.

**Study design:**

Level III, cohort study

**Results:**

At 2 months follow-up, patients performing aquatic therapy had significantly higher Constant scores (*p* < 0.001) and SSV (*p* < 0.001) compared to those performing land-based therapy or self-rehabilitation therapy. At 3 months follow-up, patients performing aquatic therapy had significantly higher Constant scores (*p* < 0.001), and SSV (*p* < 0.001), both of which exceeded the respective minimal clinically important differences (MCIDs) of 10.4 and 12. Patients performing aquatic therapy continued to have significantly higher Constant scores and SSV at 6 months, 1 year, and 2 years.

**Conclusion:**

Aquatic therapy has a positive effect on clinical outcomes at 3 months after surgery, but yields no relevant improvements on function or satisfaction at 1 to 2 years follow-up.

## Introduction

Rotator cuff tears (RCT) are among the most common shoulder injuries and can result in considerable pain and functional impairment [19]. The symptoms of RCTs can be managed conservatively by exercise therapy; however when patients do not improve or remain impaired, surgical repair is often recommended [26]. Postoperative rehabilitation following RCT repair can be of importance to restore strength, and recover normal function [8]. There is no clear consensus, however, as to whether postoperative rehabilitation achieves greater or faster recovery if supervised by a physiotherapist or self-managed by the patient at home [17, 25].

Aquatic therapy, commonly used for rehabilitation following knee and hip surgery [9, 22], or in patients with neurological conditions [3], involves performing exercises in water rather than on land. Aquatic therapy provides buoyancy to reduce body weight, which can restore physiologic muscle activation patterns by virtue of a supportive medium that attenuates forces and apprehension [1, 6]. The temperature of the water also allows relaxation and improvement of well-being, ultimately resulting in better rehabilitation. Water-based exercises can therefore allow active motion to begin at an earlier stage than land-based exercises without compromising repair integrity [2, 7].

There is increasing interest in aquatic therapy for rehabilitation following rotator cuff repair, though there is insufficient evidence regarding its efficacy [25], as comparative studies on this topic are based on small cohorts where aquatic therapy is considered as an additional component, rather than a substitute to land-based therapy [7, 25]. The purpose of this study was to compare the clinical and functional outcomes of arthroscopic rotator cuff repair over a period of 2 years using three postoperative rehabilitation modalities: aquatic therapy, land-based therapy, and self-rehabilitation therapy. The null hypothesis was that aquatic therapy would provide no difference in Constant score compared to land-based therapy or self-rehabilitation therapy.

## Methods

The authors prospectively enrolled 203 patients scheduled to undergo arthroscopic rotator cuff repair between 2012 and 2017 by the senior surgeon (BLINDED) that met the following eligibility criteria: (i) small (< 1 cm) to medium sized (1-3 cm) symptomatic supraspinatus and/or infraspinatus tendon tears, (ii) low to moderate tendon retraction according to Patte [21], and (iii) fatty infiltration stage ≤2 [11]. At the time of the preoperative assessment, patients were allocated to one of the following 3 groups based on the day of the first consultation: aquatic therapy (Tuesdays), land-based therapy (Mondays), and self-rehabilitation therapy (Wednesdays). Of the 203 patients, 188 agreed to participate and provided informed written consent for the use of their data for research. The study was approved by the local ethical committee in advance (BLINDED).

### Preoperative clinical assessment

Independent observers blinded to the study design collected patients’ age, activity level, type of work, tobacco use, as well as Constant score [5] and Subjective Shoulder Value (SSV) [10]. Rotator cuff muscle quality was assessed using computed tomography arthrography (CTA) or magnetic resonance imaging (MRI) to determine tear size according to Cofield [4], sagittal tendon retraction according to Patte [21], and degree of fatty infiltration using the modified Goutallier classification [11].

### Surgical procedure

Patients were positioned in the beach-chair position under general anesthesia and with ultrasound-guided interscalene nerve block. The arthroscopic procedure was performed using suture anchors (Mitek Healix 4.5 mm mounted with two Orthocord sutures), as well as knotless anchors for double-row fixation (Mitek Healix 5.5 mm knotless). A systematic partial bursectomy was carried out, as well as acromioplasty in cases with impingement or an offending spur [15, 16] but was limited to smoothing the bone spurs. A footprint was created and was limited to the repair size by performing gentle and superficial burring using a 5 mm Arthrex Bone Cutter. Adjuvant tenotomy or tenodesis of the long head of biceps using a 7 mm Arthrex Swivelock screw on top of the bicipital groove was performed in all patients; tenotomy was the default choice, while tenodesis was preferred for aesthetic reasons in patients < 50 years and/or women with low BMI.

Immediate post-operative care included systematic wound dressing twice per week, and stitches were removed 12 to 15 days following surgery.

### Rehabilitation protocol

Patients were immobilized for 4 weeks in a sling with the shoulder internally rotated and performed exercises at home during the immobilization phase. The exercises consisted of shoulder pendulum movements, self-assisted elbow flexion and extension, as well as gentle self-assisted passive anterior forward elevation up to 90°. In all 3 groups, patients started their rehabilitation programs after the immobilization phase of 4 weeks:

Aquatic therapy was mainly performed in a swimming pool (depth 125-140 cm, temperature 28-31 °C) supervised by a physiotherapist 2 to 3 times per week. Patients were asked to kneel or sit to submerge both shoulders to perform exercises consisting of progressive passive and active motion of the shoulder for 4-6 weeks, then additional strengthening exercises in a swimming pool for 2-4 months (Table 1).

**Table 1:** Aquatic therapy exercises

**Week 0 to 4**	Patients were immoblized in a sling but were asked to perform gentle passive motion exercises
**Week 4 to 6**	Emphasis is placed on increasing passive and active range of motion. Exercises consist of progressive passive and active motion with the shoulders submerged in water
**Week 6 to 20**	Emphasis is placed on increasing shoulder strength while continuing to increase range of motion. Exercises consist of active motion with resistance using rubber bands and water weights with the shoulders submerged in water.

Land-based therapy was performed at a rehabilitation center supervised by a physiotherapist 2 to 3 times per week. Patients performed progressive passive and active motion of the shoulder for 4-6 weeks, then additional strengthening exercises for 2-4 months.

Self-rehabilitation therapy was performed at the patient’s home without physiotherapist supervision. Patients were given a protocol, as well as additional explanations and demonstrations by a physiotherapy every 7-10 days during follow-up visits, and were instructed to perform 15–30 minutes of exercise per day. The program consisted of progressive passive and active motion of the shoulder for 4-6 weeks, then additional strengthening exercises for 2-4 months.

### Postoperative clinical assessment

Independent observers blinded to the study design collected the following outcomes at 2 months, 3 months, 6 months, 1 year and 2 years: Constant score, SSV, and patient satisfaction (0, not satisfied; 1, moderately satisfied; 2, satisfied; 3, very satisfied). All complications were recorded, such as re-tear, infection, or limited shoulder mobility (defined as a deficit of > 10° in passive range of motion in 2 planes compared to the contralateral shoulder). Patients with poor outcomes had further imaging to assess tendon healing.

### Statistical analysis

A priori power analysis was performed to determine the significance of the minimal clinically important difference (MCID) in Constant score (10.4 points) [14] among groups. Based on the findings of Mazzocca et al. [18] who reported Constant scores of 64 ± 12 at 3 months following arthroscopic repair of single-tendon rotator cuff tears, the minimum sample size required to determine the significance of the MCID with a statistical power of 0.95 was 36 patients per group. Descriptive statistics were used to summarize the data. The Shapiro–Wilk test was used to verify normality of distributions. Continuous data were compared using ANOVA or Kruskall-Wallis tests with Bonferroni correction for multiple testing. Categorical variables were compared using Chi-squared tests. *P* values < 0.05 were considered significant. Statistical analyses were performed using R, version 3.6.1 (R Foundation for Statistical Computing, Vienna, Austria).

## Results

**Fig. 1:**
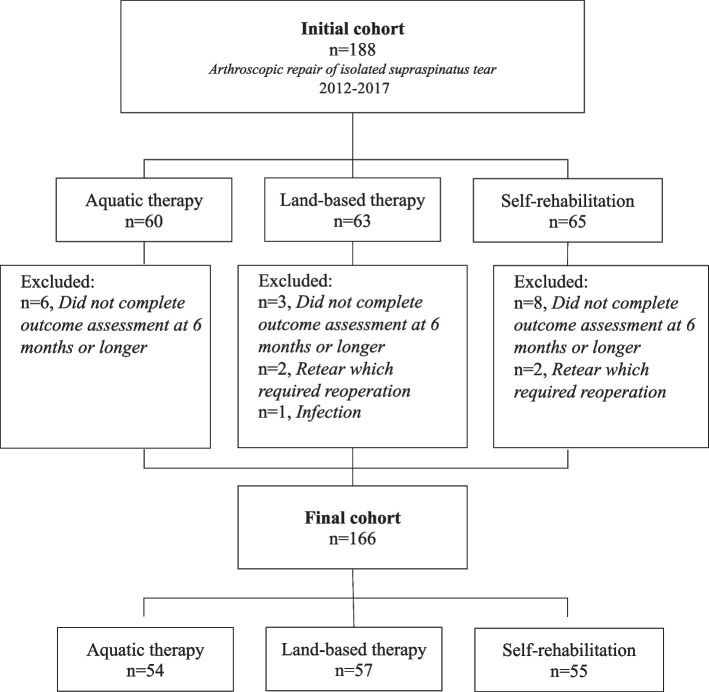
Flowchart

A total of 188 patients met the eligibility criteria; of these 5 were excluded from the final analysis because they had re-tears which required reoperation (*n* = 4, 2.1%), or deep infection (*Propionibacterium Acnes*; *n* = 1, 0.5%), and 17 (9.0%) were lost to follow-up (Fig. 1). This left a final cohort of 166 patients, of which 54 had performed aquatic therapy (33%), 57 had performed land-based therapy (34%), and 55 had performed self-rehabilitation therapy (33%). The 3 groups were similar in terms of age and proportions of men and women (Table 2).

**Table 2:** Patient demographics

	Study Cohort (***n*** = 166)			
	Aquatic (***n*** = 54)	Land-based (***n*** = 57)	Self-rehabilitation (***n*** = 55)	
	n (%) Mean ± SD *range*	n (%) Mean ± SD *range*	n (%) Mean ± SD *range*	*p value*
**Age at surgery***(years)*	56.4 ± 5.0 *(46–67)*	56.7 ± 5.5 *(47–67)*	55.3 ± 4.7 *(44–66)*	*0.410*
**Male sex**	30 (56%)	31 (54%)	28 (51%)	*1.000*
**Smoking**	11 (20%)	13 (23%)	9 (16%)	*0.894*
**Dominant side**	31 (57%)	31 (54%)	31 (56%)	*0.886*
**Biceps tenodesis**	12 (22%)	11 (19%)	10 (18%)	*0.894*
**Tear size**				*0.854*
Small	40 (74%)	42 (74%)	43 (78%)	
Medium	14 (26%)	15 (26%)	12 (22%)	
**Tendon retraction**				*0.383*
Grade 1	36 (67%)	43 (75%)	43 (78%)	
Grade 2	18 (33%)	14 (25%)	12 (22%)	
**Fatty infiltration**				*0.882*
Grade 0	7 (13%)	6 (11%)	9 (16%)	
Grade 1	30 (56%)	33 (58%)	32 (58%)	
Grade 2	17 (31%)	18 (32%)	14 (25%)	

Among the 166 patients that completed their respective therapy, 28 complications were noted (16.9%):

- 4 retears which did not require reoperation (2.4%); 2 in the aquatic therapy group, 1 in the land-based therapy group, and 1 in the self-rehabilitation therapy group (*p* = 0.779)
- 21 cases of limited shoulder mobility (12.7%); 4 in the aquatic therapy group, 8 in the land-based therapy group, and 9 in the self-rehabilitation therapy group (*p* = 0.368)
- 2 cases of minor skin rash (1.2%); both in the aquatic therapy group
- 1 superficial infection (0.6%); in the land-based therapy group

### Outcome assessment

**Fig. 2:**
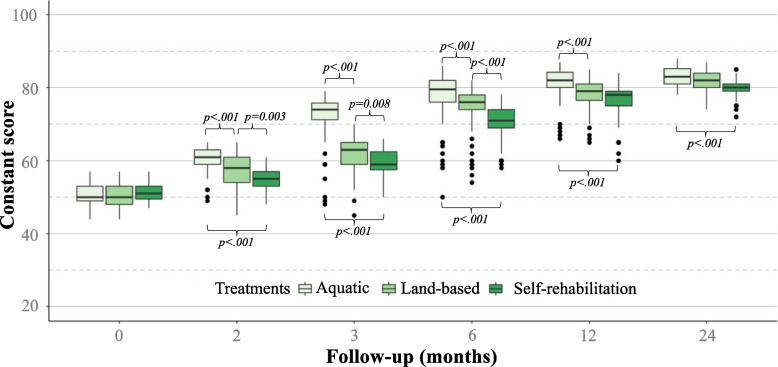
Constant score for aquatic therapy, land-based therapy, and self-rehabilitation therapy for 24 months following rotator cuff repair

**Fig. 3:**
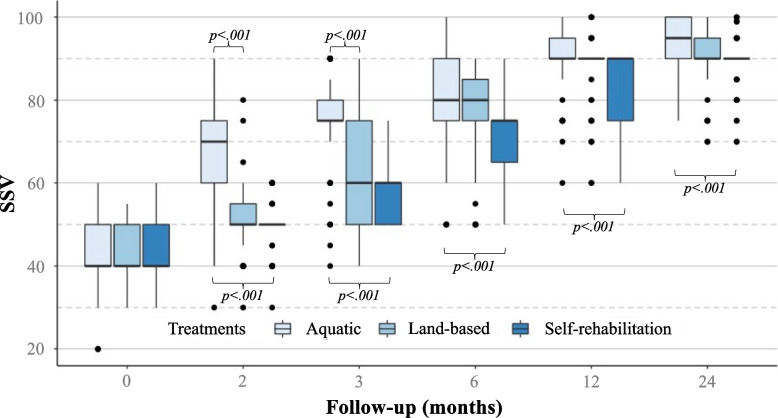
SSV for aquatic therapy, land-based therapy, and self-rehabilitation therapy for 24 months following rotator cuff repair

Preoperatively, the 3 groups had a similar Constant score (*p* = 0.245), and SSV (*p* = 0.775) (Table 3). At all follow-up evaluations, patients performing aquatic therapy had significantly higher Constant scores (*p* < 0.001) and SSV (*p* < 0.001) compared to those performing land-based therapy or self-rehabilitation therapy (Figs. 2 and 3). At 3 months follow-up, patients performing aquatic therapy had a significantly higher Constant score (*p* < 0.001), and SSV (*p* < 0.001), which exceeded the MCID of 10.4 [14], but did not exceed the minimum detectable change (MDC) of 18 points [12]. Patients performing aquatic therapy continued to have significantly higher Constant scores and SSV than land-based or self-rehabilitation therapy, at 6 months, 1 year, and 2 years, although these differences did not exceed the MCID, and therefore may not be clinically relevant.

**Table 3:** Postoperative data and clinical scores

	Aquatic (***n*** = 54)			Land-based (***n*** = 57)			Self-rehabilitation (***n*** = 55)			
	n (%) Mean ± SD	Range	95% CI	n (%) Mean ± SD	Range	95% CI	n (%) Mean ± SD	Range	95% CI	p value*
**Therapy sessions**							*NA*			
*Sessions per week*	2.5 ± 0.3	(2–3)		2.4 ± 0.4	(1–3)					
*Sessions in total*	39.0 ± 17.1	(21–90)		38.8 ± 14.2	(22–77)					
**Therapy duration***(weeks)*	16.4 ± 7.9	(9–43)		17.2 ± 10.3	(9–48)					
**Constant score***(0-100)*										
*Preoperative*	50.6 ± 3.1	(44–57)	(49–52)	50.4 ± 3.3	(44–57)	(49–52)	51.5 ± 2.4	(47–57)	(50–53)	*0.245*
*2 months*	60.3 ± 3.8	(49–65)	(59–62)	57.1 ± 4.4	(45–65)	(56–65)	54.8 ± 3.0	(48–61)	(49–65)	*<.001*
*3 months*	71.5 ± 7.5	(48–79)	(70–73)	61.7 ± 5.3	(45–70)	(60–63)	59.6 ± 3.6	(50–66)	(58–61)	*<.001*
*6 months*	77.3 ± 8.0	(50–86)	(76–79)	73.8 ± 6.8	(54–82)	(73–75)	70.6 ± 5.1	(58–78)	(69–72)	*<.001*
*1 year*	81.0 ± 5.1	(66–87)	(80–82)	78.0 ± 4.5	(65–85)	(77–79)	76.4 ± 4.9	(60–84)	(75–78)	*<.001*
*2 years*	83.4 ± 2.8	(78–88)	(82–85)	81.8 ± 3.1	(74–87)	(80–83)	79.9 ± 2.7	(72–85)	(79–81)	*<.001*
**SSV***(0-100)*										
*Preoperative*	42.5 ± 9.5	(20–60)	(40–45)	42.8 ± 7.6	(30–55)	(40–45)	43.9 ± 6.5	(30–60)	(41–46)	*0.775*
*2 months*	68.4 ± 11.8	(30–90)	(66–71)	51.1 ± 8.6	(30–80)	(49–54)	49.6 ± 6.2	(30–60)	(47–52)	*<.001*
*3 months*	74.9 ± 12.1	(40–90)	(72–77)	63.6 ± 12.1	(40–90)	(61–66)	58.6 ± 8.2	(50–75)	(56–61)	*<.001*
*6 months*	80.8 ± 12.4	(50–100)	(78–83)	76.5 ± 12.1	(50–90)	(74–79)	71.4 ± 9.3	(50–90)	(69–74)	*<.001*
*1 year*	89.9 ± 8.8	(60–100)	(87–92)	87.4 ± 8.8	(60–100)	(85–90)	82.3 ± 9.5	(60–90)	(80–85)	*<.001*
*2 years*	92.7 ± 7.5	(75–100)	(90–95)	91.1 ± 7.6	(70–100)	(88–93)	88.9 ± 6.5	(70–100)	(86–91)	*<.001*
**Satisfaction***(0, not satisfied-3, very satisfied)*										
*2 months*	1.6 ± 0.6	(0–2)	(1.38–1.73)	1.2 ± 0.6	(0–2)	(0.99–1.33)	0.7 ± 0.6	(0–2)	(0.50–0.85)	*<.001*
*3 months*	2.0 ± 0.8	(0–3)	(1.79–2.14)	1.6 ± 0.8	(0–3)	(1.41–1.75)	1.2 ± 0.8	(0–3)	(1.05–1.39)	*<.001*
*6 months*	2.3 ± 0.7	(0–3)	(2.12–2.47)	2.1 ± 0.7	(0–3)	(1.94–2.27)	2.0 ± 0.7	(0–3)	(1.83–2.17)	*0.083*
*1 year*	2.5 ± 0.6	(1–3)	(2.34–2.69)	2.3 ± 0.5	(1–3)	(2.15–2.50)	2.2 ± 0.6	(1–3)	(2.06–2.41)	*0.065*
*2 years*	2.6 ± 0.6	(1–3)	(2.43–2.78)	2.6 ± 0.5	(1–3)	(2.41–2.75)	2.6 ± 0.5	(2–3)	(2.42–2.77)	*0.960*

*Abbreviations*: *SSV* Subjective Shoulder Value, *CI* Confidence Interval

**Fig. 4:**
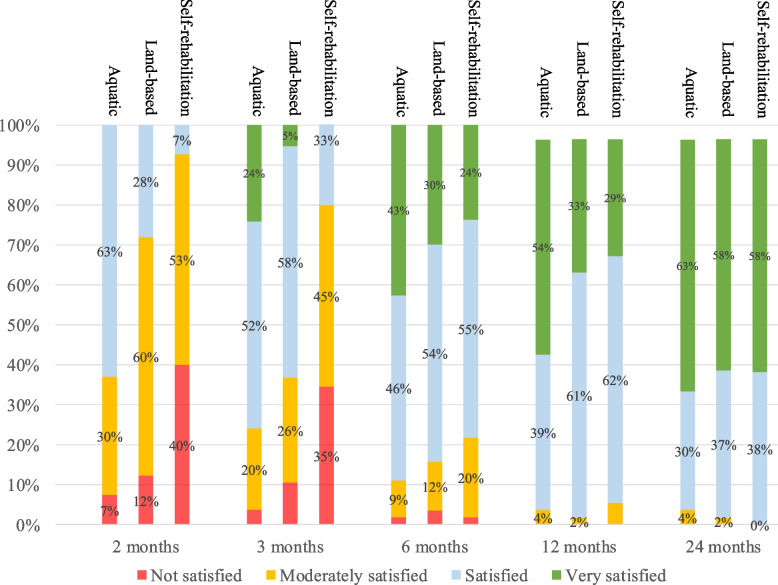
Satisfaction for aquatic therapy, land-based therapy, and self-rehabilitation therapy for 24 months following rotator cuff repair

Patients that performed aquatic therapy rated their overall satisfaction significantly higher (*p* < 0.001) than those that performed land-based therapy or self-rehabilitation therapy, at 2 and 3 months, while their overall satisfaction was similar to the 2 other groups at 6 months (*p* = 0.083), 1 year (*p* = 0.065), and 2 years (*p* = 0.960) (Fig. 4).

## Discussion

The most important findings of this study were that, at 3 months following arthroscopic rotator cuff repair, patients performing aquatic therapy had clinically significant higher Constant scores, that exceeded the MCID, compared to those performing land-based therapy or self-rehabilitation therapy, but yields no relevant improvements in function or satisfaction at 1 to 2 years follow-up The difference in Constant score did not exceed the MDC at any timepoint. These findings confirm the hypothesis that aquatic therapy would provide faster improvement in Constant score than land-based therapy or self-rehabilitation therapy.

A recent systematic review by Thomson et al. [25] compared exercise therapies, use of continuous passive motion (CPM), and duration of postoperative immobilisation to determine the most effective rehabilitation protocol following rotator cuff repair. The authors suggested that following the repair, patients should expect improvement in pain, ROM and function, but there was no benefit in favour of a particular rehabilitation method. It is worth noting, however, that the review of Thomson et al. did not include aquatic therapy.

Conventional land-based rehabilitation has for long been the standard choice following rotator cuff repair; however, the present study suggests that aquatic therapy may provide slightly faster and better recovery. The findings of the present study corroborate the feasibility study by Brady et al. [1] who found that aquatic therapy is associated with minimal risk and is worthy of investigation in future clinical trials. Low-stress aquatic exercises, performed both in the clinic and at home or in a local gym, can be a practical and valuable addition to the process of recovery from rotator cuff repair [13, 23]. The added benefits of exercising in a warm, buoyant environment early in the recovery process has proven to increase psychological and physiological well-being [2]. The assessment of self-image and confidence in abilities is an often overlooked but important aspect of recovery, and could play a role early in the healing process [2]. Compared to ambient air temperature, immersion at 32 °C has been found to lower the heart rate by 15%, systolic blood pressure by 11% and diastolic blood pressure by 12% [20, 24]. In addition, Kelly et al. [13] found that shoulder elevation in water resulted in significantly lower activation of the rotator cuff and synergistic muscles. The decreased muscle activation during aquatic physical therapy allows for earlier active motion in the postoperative period without compromising patient safety.

The results of the present study must be interpreted with the following limitations in mind. First, the authors are aware that assigning patients in a rehabilitation group according to the day of the week does not constitute true randomization as per current standards, which were unknown to the authors prior to the start of the study 10 years ago. Therefore, despite similar preoperative characteristics among the 3 groups, selection bias could have been introduced by assigning patients to a treatment based on the consultation day. Second, the authors did not record all preoperative tear characteristics and tissue quality, which could influence improvements in clinical scores. Third, there was some heterogeneity in the adjuvant procedures, which were performed in similar proportions among the three groups but could still introduce bias. Finally, the study did not investigate the factors that contribute to the differences in clinical scores among the three groups, which may be physiological or psychological. Future studies could investigate this further, notably by collecting the SF-12 scores at each time point, as it distinguishes between physical and mental health.

## Conclusion

Aquatic therapy has a very limited positive effect on clinical outcomes at 3 months after surgery, but yields no relevant improvements on function or satisfaction at 1 to 2 years follow-up.

## Data Availability

Data is available upon suitable request.
