# Correction: Aquatic therapy following arthroscopic rotator cuff repair enables faster improvement of Constant score than land‑based therapy or self‑rehabilitation therapy

**DOI:** 10.1186/s40634-023-00602-2

**Published:** 2023-04-04

**Authors:** Alec Cikes, Fayssal Kadri, Floris van Rooij, Alexandre Lädermann

**Affiliations:** 1 Synergy Medical Centre, Medbase Group, Lausanne, Switzerland; 2 Hirslanden, Bois Cerf Clinic, Lausanne, Switzerland; 3 ReSurg SA, Rue Saint Jean 22, 1260 Nyon, Switzerland; 4 Division of Orthopaedics and Trauma Surgery, La Tour Hospital, Meyrin, Switzerland; 5 Faculty of Medicine, University of Geneva, Geneva, Switzerland; 6 Division of Orthopaedics and Trauma Surgery, Department of Surgery, Geneva University Hospitals, Geneva, Switzerland

**Correction: J Exp Ortop 10, 2 (2023)**

10.1186/s40634-022-00554-z

Following publication of the original article [1], the authors identified an error in the conclusion section of the Abstract.

The conclusion of the abstract section states:

“Aquatic therapy has a very limited positive effect on clinical outcomes at 3 months after surgery, but yields no relevant improvements on function or satisfaction at 1 to 2 years follow-up.”, which is in contradiction with the conclusion in the article itself.

This text should be changed to:

Aquatic therapy has a positive effect on clinical outcomes at 3 months after surgery, but yields no relevant improvements on function or satisfaction at 1 to 2 years follow-up.

The original article [1] has been corrected.
